# Metagenomic Insights into Pathogenic Characterization of ST410 *Acinetobacter nosocomialis* Prevalent in China

**DOI:** 10.3390/pathogens11080838

**Published:** 2022-07-27

**Authors:** Liang Jing, Zhuofei Xu, Youping Zhang, Dayong Li, Yaqin Song, Hongjie Hu, Yuan Fang, Wei Zhu

**Affiliations:** 1 Department of Emergency-Critical Medicine, Tongji Hospital, Tongji Medical College, Huazhong University of Science and Technology, Wuhan 430030, China; jingl7929@gmail.com (L.J.); zhangyouping2022@126.com (Y.Z.); dayongli1989@163.com (D.L.); 18772954675@163.com (Y.S.); hhj521520@163.com (H.H.); 2 Genoxor Medical Science and Technology Inc., Zhejiang 317317, China; zhuofei.xu@genoxor.com

**Keywords:** *Acinetobacter nosocomialis*, opportunistic pathogen, metagenomic surveillance, pathogenic risk, genome assembly, phylogeny, ST410, virulence-associated genes

## Abstract

*Acinetobacter nosocomialis* is a prevalent opportunistic pathogen that causes hospital-acquired infections. The increasing threats from *A. nosocomialis* infections have led to attention from the scientific and medical communities. Metagenomic next-generation sequencing (mNGS) was performed for an exudate specimen collected from an ICU patient with wound infection, followed by sepsis, in Tongji Hospital. Three assembly strategies were employed to recover the genome of *A. nosocomialis* in the metagenomic sample. Together with publicly available genomes of *A. nosocomialis*, the features of population genetics and molecular epidemiology were deeply analyzed. A draft genome was reconstructed for the metagenomic strain WHM01, derived from the ST410 *A. nosocomialis* dominating the microbial community, thereby prompting its highly pathogenic risk, which is associated with infection and persistence. The structure of the bacterial pangenome was characterized, including the 1862 core and 11,815 accessory genes present in the 157 strains. The genetic diversity of the genes coding for the 128 virulence factors assigned to 14 functional categories was uncovered in this nosocomial pathogen, such as the lipooligosaccharide, capsule, type IV pilus, and outer membrane proteins. Our work revealed genomic properties of ST410 *A. nosocomialis*, which is prevalent in China, and further highlighted that metagenomic surveillance may be a prospective application for evaluating the pathogenic characteristics of the nosocomial opportunistic pathogens.

## 1. Introduction

Gram-negative bacterium *Acinetobacter nosocomialis* belonging to gamma-proteobacteria is a common nosocomial opportunistic pathogen within the *A. calcoaceticus-baumannii* (ACB) complex. The ACB complex includes the other four medically important bacteria *A. baumannii*, *A. pittii*, *A. seifertii*, and *A. lactucae*, as well as a nonpathogenic soil microorganism *A. calcoaceticus* [1,2]. Since *Acinetobacter* spp. share indistinguishable phenotypes, conventional biochemical tests often misidentify bacterial isolates at the species level. During the last decade, molecular methods have become mature for accurate species identification, e.g., multilocus sequence typing (MLST) [3], whole-genome sequencing [4], and mass spectrometry [5]. From the viewpoint of global epidemiology, *A. baumannii* is the most predominant species isolated from human specimens and responsible for more than 80% of the infections caused by the ACB complex [6,7,8]. However, the prevalence of *A. nosocomialis* infections has been rising in recent years. In 2019, Chen et al. reported that the proportion of *A. nosocomialis* increased by 6.4% among 1041 ACB complex strains isolated between 2010 and 2014 in China [9]. MLST analysis has pointed out that ST71 *A. nosocomialis* isolates are related to the dissemination of bacteria in the Netherlands [3] and Brazil [10]. In China, the frequently isolated types are ST410, ST68, and ST1272 [11]. Hospital-acquired infections caused by *A. nosocomialis* often lead to bacteremic pneumonia and bloodstream infection, particularly among the immunocompromised patients and those with indwelling devices [12,13].

The increasing threats from *A. nosocomialis* infections in the healthcare setting have been paid more attention by the scientific and medical communities. To exploit the disease-causing mechanisms of this clinically relevant pathogen, the recent experimental investigation into the bacterial surface components has shown the important roles of several virulence factors in the pathobiology of *A. nosocomialis* [14]. For instance, type IV pili (T4P) and its glycosylation phenotype in *A. nosocomialis* strain M2 have been characterized by their abilities for twitching motility and natural transformation [15]. Another study has revealed that *A. nosocomialis* harbors a functional type II secretion system that plays a role in exporting effector proteins out of the cell, such as the lipases LipA and LipH and metallopeptidase CpaA [16]. Besides, Cosgaya et al. have verified the virulence potential of the five species within the Ab group (*A. baumannii*, *A. nosocomialis*, *A. pittii*, *A. seifertii*, and *A. lactucae*) using in vitro and in vivo models [2]. However, the systemic investigation of the genic repertoire associated with virulence functions is still needed for elucidating the multifactorial nature of *A. nosocomialis* pathogenicity.

On the other hand, *A. nosocomialis* is opportunistically pathogenic and possesses the capability of persistent infection in the hospital environment, thus posing a challenge for accurate diagnosis. Currently, the rapid development of the metagenomic next-generation sequencing (mNGS) technique has provided valuable approaches to capture the ecological changes of the potential pathogens in the dynamic microbial community of various niches [17]. The mNGS testing has enabled researchers to identify and quantify any microbes in a sample, e.g., an enteroaggregative *Escherichia coli* strain O104:H4 detected in the metagenomes from the 2011 Germany outbreak [18]. More recently, a sputum microbiome study utilizing the mNGS data has established a microbial abundance catalog of species-level detection limits for evaluating pathogenic risk, which is especially useful for inferring infection and/or the colonization of opportunistic pathogens in the community [19].

Here, we identified an *A. nosocomialis* strain showing a highly pathogenic risk for an ICU-admitted patient, based on the metagenomic data. To reconstruct the genome of the pathogen target, three assembly strategies were adopted and compared. Phylogenomic analysis was performed to characterize the epidemiological features of the metagenome strain. The genetic diversity of the virulence functions was investigated to better understand the pathogenesis of this human opportunistic pathogen.

## 2. Materials and Methods

### 2.1. Specimen Collection

In this study, wound exudate was sampled from a 62-year-old male with shock symptoms of dyspnea and hemodynamic instability in Tongji Hospital (Wuhan, China), in May of 2021. The patient had injured his left lower limb, due to falling two months earlier, and then experienced extensive lesions of skin ulceration, fatigue, and anorexia in the following weeks. Additionally, he had a history of diabetes. Upon admission to the intensive care unit (ICU), the lesions were initially diagnosed with sepsis, multiple organ dysfunction syndromes, septic shock, skin, and soft tissue infections. Based on the routine microbiological culture, *Streptococcus pyogenes* was identified from the pyogenic fluids of the wound site. Meanwhile, the wound sample was transported in a package with dry ice cooling agents for metagenomic sequencing.

### 2.2. Metagenomic Sequencing and Taxonomic Classification

The sample was preprocessed through centrifugation to remove the supernatant, and the pellets were used for DNA extraction using the HostZERO^TM^ Microbial DNA Kit (Zymo Research, Irvine, CA, USA). Approximately 10 ng DNA was used as input for library construction using Hieff NGS OnePot Pro DNA library prep kit (Yeasen Biotech., Shanghai, China). Total genomic DNA was fragmented into ~300 bp inserts, followed by adding Illumina Trueseq adaptors. The quality of the library was then assessed with a Qsep100 instrument (Bioptic Inc., New Taipei City, China). The sequencing experiment was performed using the Illumina NovaSeq 6000 platform to generate 150-bp paired-end reads at Novogene, Beijing, China. Quality trimming and filtering of the raw reads were conducted using TruSeq adapter sequences and the program Fastp v0.21.1 [20]. Host-derived reads were removed by mapping to the human reference genome GRCh38 using Bowtie v2.2.6 [21]. To quantify the relative abundances of individual species, taxonomic profiling was estimated by Kraken v2.0.9 [22]. The most abundant species in the community was identified to be *A. nosocomialis*, with 7,213,985 reads.

To evaluate the putative pathogenic risk in the patient’s specimen, we retrieved the abundance values of *A. nosocomialis* in the healthy human microbiomes of five body sites, according to the curated metagenomic database implemented by the R package curatedMetagenomicData [23]. The numbers of samples for each site were 363 samples for skin, 740 samples for oral, 93 samples for nasal cavity, 12,998 samples for stool, and 96 samples for vagina. The presence of *A. nosocomialis* was detected in the three sites, and the percentage abundances per sample are illustrated in Figure S1.

### 2.3. Genome Assembly of the Targeted Pathogen

Next, we employed three strategies (designated as Assembly I–III hereafter) for the genomic reconstruction of *A. nosocomialis* from metagenomic reads. For Assembly I, the reads assigned to the ACB complex (NCBI TaxID: 909768) were extracted according to the Kraken classification with default options [22]. The extracted reads were used for *de novo* assembly using Spades v3.15.4 with the options -t 24 -m 128 --cov-cutoff auto --isolate [24]. For Assembly II, metagenome assembly was performed using MetaSpades v3.15.4, and genome binning was then carried out using the binning and bin_refinement modules in the package metaWRAP v1.3.2 [25]. For Assembly III, the RefSeq genomes of 156 *A. nosocomialis* isolates were retrieved from the NCBI Assembly database in January of 2022. The recruitment of the metagenomic reads affiliated to *A. nosocomialis* was performed by BBmap v38.18 [26], and the captured reads were assembled using Spades. The quality assessment of the resulting genome assemblies was conducted by both QUAST v5.0.2 [27] and CheckM v1.0.18 programs [28].

### 2.4. Analysis of Phylogenome and Pangenome

Using FastMLST v0.0.15 [29], MLST was conducted, based on the Pasteur scheme (abaumannii#2), with seven conserved alleles of *A. nosocomialis*: *cpn60*, *fusA*, *gltA*, *pyrG*, *recA*, *rplB*, and *rpoB* [3]. Genome-wide average nucleotide identity (ANI) between any strain pairs was calculated by using PYANI v0.2.11, with the option -m ANIb [30]. Species identification of bacterial genomes was also performed by using the approach of ribosomal MLST implemented by the BIGSdb platform [31,32]. The package Snippy v4.4.0 [33] was used to produce a sequence alignment of core SNPs across all the genomes. Subsequently, a maximum likelihood phylogenomic tree was built using FastTree v2.1.10 with the generalized time-reversible model [34]. The pairwise SNP distance matrix between any two genomes was calculated by snp-dists v0.6.3. In addition, the MLST phylogenetic tree was constructed according to the alignment of seven gene loci by Muscle [35] and FastTree. Integration of the phylogeny with other associated data was visualized using GGTREE v3.2.1 [36].

For the pangenome analysis, gene calling of open-reading frames was performed for the genome reconstructed above and public isolate genomes of *A. nosocomialis* using Prokka v1.14.6 [37]. Detection and clustering of the orthologous genes (OG) were carried out using Roary v 3.13.0, with the options -p 36 -i 90 -e -n -t 11 -s -cd 100 -a -v [38]. A codon-aware alignment of all the core genes was generated using PRANK v.170427 [39]. A phylogenetic tree was then built by FastTree v2.1.10. Amino acid sequences of the representative genes from each OG were extracted and annotated, according to the best hits and *e*-value threshold of 1e^−20^, by Blastp v2.9.0+, searching against the UniRef50 database [40]. Protein structure domains were predicted by hmmscan v3.2.1, searching against Pfam v32 [41]. The genes encoding virulence factors (VFs) were detected by Blastp against the VFDB database, which contains 4165 genes associated with experimentally verified VFs [42]. The putative virulence genes were screened out, based on the *e*-value threshold of 1e^−20^, and further used as gene markers to investigate their distribution across all the strains using the package LS-BSR v1.2.3 [43].

## 3. Results

### 3.1. Pathogenic Risk of A. nosocomialis

In this study, we initially found that more than half (51.2%) of all microbial reads (14,093,277) were assigned to *A. nosocomialis* through the mNGS testing on wound exudate from an ICU patient with septic shock. The taxonomic profiling showed that 42 microbial species, with a relative abundance above 0.1%, were present in the community (Table S1). Of these taxa, 11 *Acinetobacter* species were predicted, and the abundance of *A. nosocomialis* was estimated to be 66.5%. Except for *A. nosocomialis*, the abundances of the other species ranged from 4.4% for *A. baumannii* to 0.1% for *A. haemolyticus*. Interestingly, *S. pyogenes* was the single species detected by conventional culture in the hospital setting, but low abundance (0.024% and 2778 reads) was found for these gram-positive streptococci, according to the mNGS testing. To provide a reference for prompting abnormal microbial composition, we also retrieved the abundances of *A. nosocomialis* present in >10,000 samples from the healthy human microbiomes using public metagenomic resources (Table 1). The results show that *A. nosocomialis* is sometimes present (4.96%) in the microbiota of the skin and rarely present in the microbiota of oral and stool. Moreover, *A. nosocomialis* exhibits low abundance in the normal flora, with the maximum estimate of 0.4% and 0.8% observed in skin and stool, respectively (Figure S1). Based on the remarkably high abundance of *A. nosocomialis* dominating the community of the specimen, it indicated that *A. nosocomialis* was a high-risk pathogen associated with the infection in the case.

### 3.2. Comparison of Three Assembly Strategies

To uncover the characterizations of the pathogenic *A. nosocomialis* strain in the metagenomic sample, we reconstructed its genome using three assembly strategies. The quality metrics of the resulting genome assemblies are summarized in Table 2. According to the total contig size and N50, the best genome assembly was the one generated by the Assembly III (reference genome-based read recruitment, followed by *de novo* assembly), including 123 contigs more than 500 bp, total contig size of ~3.89 Mb, and N50 of 82,591 bp. The percentages of genome completeness and contamination were 100% and 0%, respectively. Besides, the assembly metrics generated by the Assembly II (metagenomic assembly and binning) were also good, resulting in 103 contigs (>500 bp), a genome size of ~3.87 Mb, and N50 of 79,429 bp. The genome quality generated by the Assembly I (Kmer-based read classification and assembly) was relatively poor, with N50 of 40,596 bp. Based on the above evaluation, the genome of Assembly III with a GC content of 38.77% was used for the subsequent phylogenomic and pangenomic analyses.

### 3.3. Phylogeny of the Genome from the Assembly III

**Figure 1:**
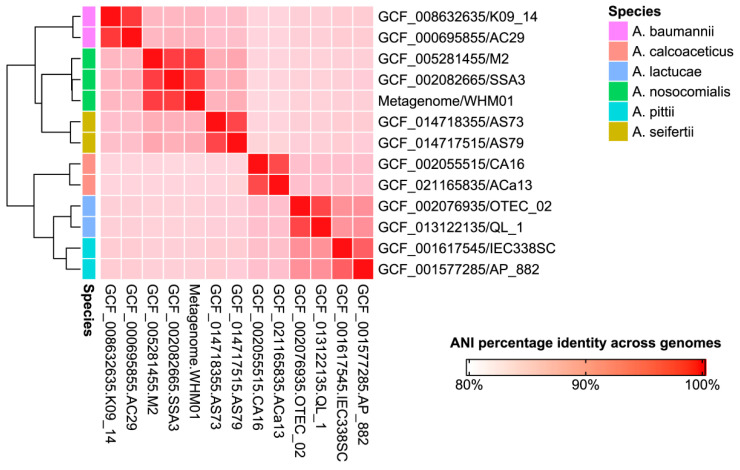
Species identification for the genome of *A. nosocomialis* strain WHM01 recovered from the clinical metagenome. The heatmap shows the color-coded ANI values between each pair of genomes from the six species within the genus *Acinetobacter*. For each species, two complete genomes retrieved from NCBI Assembly database were used for the comparative analysis.

The analysis of genome-wide nucleotide sequence identity was first performed to infer the organismal origin of the metagenome strain (designated as WHM01 hereafter). As shown in Figure 1, WHM01 shared 97.64% and 97.63% ANI with both genomes of *A. nosocomialis* strains M2 and SSA3, respectively, while the genome of WHM01 possessed relatively low ANI values with the genomes from the other *Acinetobacter* species, ranging from 86.23% to 91.92% (Table S2). Since the 95% ANI threshold can demarcate species boundaries [4], it again confirmed that the strain WHM01 belonged to *A. nosocomialis*. Species demarcation of all the genomes used for the subsequent comparison was further verified, based on the rMLST approach (Table S3). Using the Pasteur MLST scheme for *A. nosocomialis*, the sequence type of WHM01 was determined to be ST410 (20-26-26-14-26-16-23).

**Figure 2:**
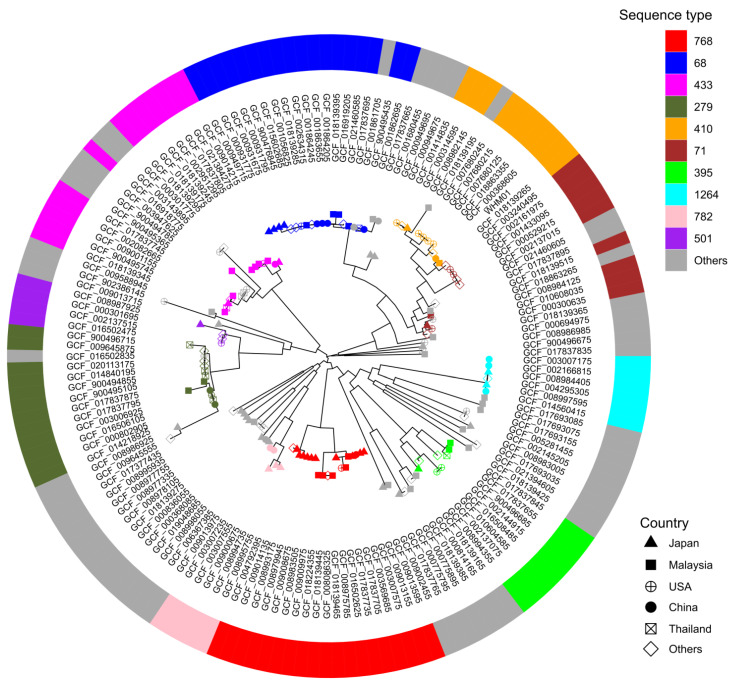
Maximum likelihood phylogeny of *A. nosocomialis*. The phylogenetic tree was built based on 169,300 core SNPs of 157 *A. nosocomialis* genomes and surrounded by a colored ring representing sequence types of the corresponding strains. The shapes of the tip nodes of the tree stand for the countries in which the strains are collected.

To explore the relationships between WHM01 and 156 *A. nosocomialis* isolates with public genome data, a phylogenetic tree based on a core-genome SNP alignment was reconstructed and integrated with the bacterial sequence types in Figure 2 (see more details in Table S3). The SNP distance matrix of pairwise genomes of *A. nosocomialis* is shown in Table S4. Among all the strains, 33 STs were detected, and 13 strains were assigned to the novel STs. The top five prevalent STs were ST768 (19 strains), ST68 (18), ST433 (13), ST279 (12), and ST410 (10), respectively. The strain WHM01 was most closely related to the ST410 strain AC1892 (GCF_018139265) isolated in Malaysia, 2018. The genetic distance between the strains WHM01 and AC1892 was 206 SNPs. In terms of geographical distribution, the top five countries were Japan (39), Malaysia (37), America (22), China (17), and Thailand (10) (Figure 2). The ten strains belonging to ST410 were sampled from America (5), the Czech Republic (1), Iraq (1), Malaysia (1), Japan (1), and China (1). Meanwhile, the topology of the strain tree of *A. nosocomialis* was also investigated, based on the sequence alignments of core genes present in all the genomes and seven MLST loci, respectively. A total of five major evolutionary branches were observed and consistent between the core SNP (Figure S2A) and core genome trees (Figure S2B), whereas the corresponding topological pattern was inconspicuous in the MLST-based tree (Figure S3). The strains belonging to the same STs tended to be clustered together, e.g., ST768 (19), ST395 (8), ST1264 (6), and ST782 (5). Clade IV was exclusively composed of the ST768 strains. The strains of ST410 were clustered into the branch of Clade I and closely related to the strains of ST71. Notably, the subclade of ST410 strains encompassed a strain (GCF_018139195) that was assigned to a novel ST (20-24-26-14-26-16-23).

### 3.4. Virulence-Associated Genes in the Pangenome of A. nosocomialis

To investigate the genetic content of genes coding for virulence factors, we carried out a pangenome analysis using 578,446 protein-coding sequences (CDSs) across the 157 genomes of *A. nosocomialis*. Gene clustering resulted in 13,677 OG clusters, which can be separated into two gene sets: the 1862 core OGs present in all the strains and remaining ones, as the accessory genes present in at least one strain, but not all. Among all the OGs, 3742 were strain-specific genes that may be associated with unique phenotypes of individual strains. The functional annotations of all OGs are recorded in Table S5.

**Figure 3:**
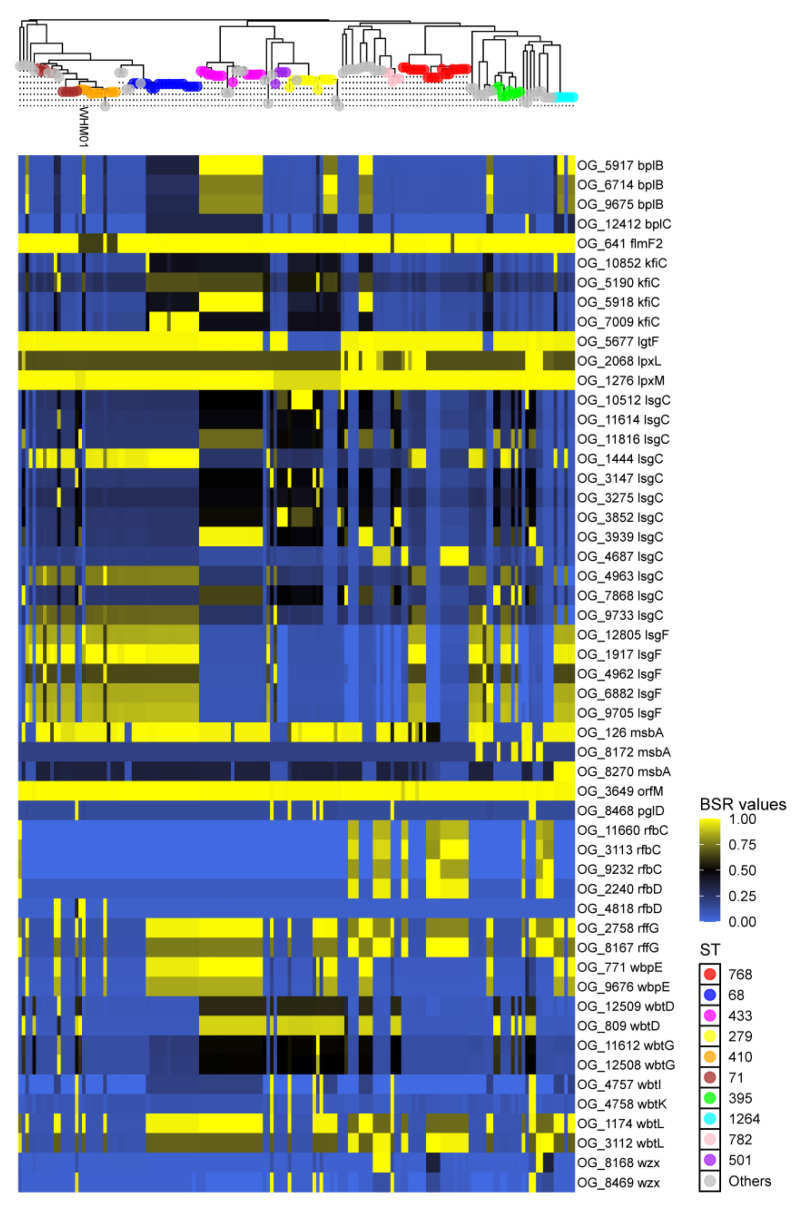
Genetic distribution and sequence conservation of selected accessory genes involved in the biosynthesis of LOS in *A. nosocomialis*. The upper tree is the whole genome SNP-based phylogeny shown in Figure 2. The colored tip nodes correspond to different STs. Sequence conservation of virulence-associated gene markers are color-coded according to the BLAST score ratio values summarized in Table S7.

Next, 759 virulence-associated genes (VAGs) were detected in the pangenome of *A. nosocomialis*. About two-thirds (531 genes) of all the VAGs were accessory genes, and the remaining were core genes. The detected VAGs encode the products functioning in 128 virulence factors affiliated with 14 major functional classes, such as immune modulation (137 genes), effector delivery systems (115), adherence (86), exotoxins (48), biofilm (36), and exoenzymes (2) (Table S6). Among the VAGs detected, 426 genes were encoded in the genome of WHM01, including 228 core genes and 198 accessory genes. Some core genes of *A. nosocomialis* were found to be well-associated with the virulence factors whose chemical domains are highly conserved in Gram-negative bacteria, e.g., lipid A of lipopolysaccharides (LPS) [44] and type IV pili (T4P) [45]. The genes involved in the biosynthesis of LPS lipid A were uniformly present in all *A. nosocomialis* strains, including *fabZ*, *flmK*, *hisH2*, *kdsA*, *kdtB*, *lpsB*, *lpxABCDHKL*, *msbA*, *waaA*, and *wbuZ* (Table S6). Besides, 58 genes involved in T4P synthesis were identified in the pangenome of *A. nosocomialis*, including 17 core and 41 accessory genes. The substantial number of accessory VAGs may contribute to diversified phenotypes and virulence of individual strains. Figure 3 and Figures S4–S6 display the mosaic patterns and genetic divergence of the accessory genes coding for cell surface virulence factors in *A. nosocomialis*, such as LPS, capsule, T4P, biofilm, and exotoxins. The genetic diversity of the VAGs was discussed in more detail below.

## 4. Discussion

As is well-known, *Acinetobacter* infections frequently occur in the inpatients receiving treatment in the healthcare environment [46]. Among the prevalent *Acinetobacter* species, *A. baumannii* is the primary nosocomial pathogen that has been intensively studied on its ecology, epidemiology, and pathogenic mechanisms [46,47]. In comparison to *A. baumannii*, *A. nosocomialis* is another distinct clinical entity whose medical importance has been increasingly reported, due to the high incidence in the population of hospital-acquired infections [13]. To further understand the pathogenesis of this emerging pathogen in research and clinical settings, we established a framework supporting the investigation from metagenomics to population genomics in the present study, and the relevant results were discussed and compared with *prior* knowledge of *A. nosocomialis*.

### 4.1. Clinical Significance of Metagenomic Surveillance on Opportunistic Pathogen

Routine culture and biochemical testing are likely to misidentify certain pathogens implicated in infectious diseases, due to limited conditions in the hospital setting [48]. Murni et al. have reported that the blood culture contaminants frequently detected from the specimens often involve opportunistic pathogens, such as coagulase-negative staphylococci, *Streptococcus* spp., and *Pseudomonas* spp. [49]. The growth of bacterial pathogens from certain taxonomic lineages require more specific culture media and the addition of indispensable nutritional ingredients [50]. For instance, nicotinamide-adenine-dinucleotide (NAD) is an essential growth factor for some clinically relevant pathogenic species within the genus *Haemophilus* [51]. Particularly, the growth of *H. influenzae* normally needs both hemin and NAD at 35–37 °C, with ~5% CO_2_. In practice, the clinical culture would adopt several commonly used, but not many, types of media for isolation of non-fastidious bacteria, which may miss many organisms with stringent nutritional requirements for culture in vitro. In our study, Gram-positive *S. pyogenes* was the sole species cultivated and reported by culture in the hospital. In comparison to conventional culture, mNGS may provide an ecological landscape of microbial composition in the specimen from the wound infection. Here, *S. pyogenes* was also detected by mNGS, with about two thousand reads accounting for low abundance in the community, while the abundance of *A. nosocomialis* in the specimen from the diseased patient was far greater than that in the healthy human microbiome, thereby indicating that Gram-negative *A. nosocomialis* was a high-risk pathogen implicated in the lesions of the patient. Since *A. nosocomialis* is a nosocomial opportunistic pathogen, it is insufficient to determine whether the bacterial strain is pathogenic or not based on the single evidence provided from species identification. The mNGS testing can provide auxiliary information on the percentage abundance of the individual species for robust distinguishing between the pathogenic and commensal/nonpathogenic strains.

### 4.2. Genetic Diversity of Virulence Factors

VFs localizing at the bacterial surface play vital roles in the interactions with the host immune system, transport of molecules into and out of the cell, and protection from external stresses for pathogenic *Acinetobacter* spp. [14]. The structural diversity of the VFs in the distinct strains/species is usually determined by the rapidly evolving genic sequences under natural selection or host immunological pressure. Therefore, we next investigated the genetic variation of several VFs associated with colonization, persisting infection, and biofilm formation of *A. nosocomialis*, such as lipooligosaccharide (LOS), capsule, adhesins, pili, and outer membrane proteins.

LPS (also termed endotoxin), a primary structural component on the Gram-negative outer member, can trigger the host inflammatory response and further induce sepsis and septic shock among immunocompromised patients [52]. A classical LPS consists of an endotoxic lipid A, core oligosaccharide, and repeating sugar structure, called the O-antigen chain. Most *Acinetobacter* spp. have been considered to produce LOS, which is a specific kind of LPS without an O-antigen [14]. In general, the complex sugar moieties of LOS are synthesized by a set of diverse enzymes bearing great genetic variations. Here, a repertoire of 78 genes encoding enzymes involved in the LOS biosynthesis pathway was identified in the pangenome of *A. nosocomialis* (Table S6). As shown in Figure 3, the majority of the accessory genes exhibited remarkable sequence diversification across the strains, especially the genes coding for glycosyltransferases. For instance, 12 variants of *lsgC* encoding group 1 family glycosyltransferases were detected, all of which possessed both Pfam domains Glycos_transf_1 (PF00534) and Glyco_trans_1_4 (PF13692). Besides, four variants (OG_5918, OG_7009, OG_10852, and OG_5190) of *kfiC* encoding family 2 glycosyltransferases were found to be present in the 22, 13, 3, and 2 strains, respectively (Table S6). High numbers of gene variants, again, confirm the previous option that a variety of glycosyltransferases enable the production of highly variable structures of LOS core, thus constituting the foundation of serotyping schemes for the strains of the same species [14]. Additionally, a *pglL*-like core gene (OG_288) encodes an *O*-oligosaccharyltransferase (*O*-OTase) enzyme (542 aa), which shares 81.3% identity with a homology (472 aa) of *A. baumannii* (Table S5). *O*-OTase of *A. nosocomialis* encompasses three classical domains, i.e., Wzy_C (PF04932), Wzy_C_2 (PF11846), and PglL_A (PF15864), which are involved in the *O*-lined protein glycosylation that can exert pleiotropic effects on bacterial survival and biofilm formation [53].

Like LOS, capsular polysaccharide (CPS) is another active surface glycoconjugate participating in bacterial immune evasion and virulence of pathogenic *Acinetobacter* spp. The pangenome of *A. nosocomialis* possessed 47 genes (12 core genes and 35 accessory genes) encoding the enzymes responsible for CPS polymerization, assembly, and transport to the cell surface (Table S6). Among the set of core genes, some encode the enzymes essential for bacterial capsule biogenesis, e.g., OG_1850 sharing 62.7% amino acid sequence similarity with a capsule assembly protein Wzi of *Klebsiella pneumoniae* [54], and OG_94 sharing 43.3% similarity with *Bacillus anthracis* CapA [55]. Additionally, three variants (OG_1133, OG_3853, and OG_2122) of *wza* encoding an outer membrane polysaccharide transporter were identified, all of which harbor the domain Poly_export (PF02563) functioning in the CPS export. All the Wza proteins of *A. nosocomialis* are homologous to *A. baumannii* EpsA, which is critical for the capsule-positive phenotype [56]. It was apparent that all the strains were just divided into three groups, according to the *wza* variants: OG_1133 present in 130 strains, OG_3853 in 16 strains, and OG_2122 in 11 strains (Figure S4).

Bacterial adhesion to the surface of mucosal epithelial or endothelial cells is essential for the colonization and infection of many pathogenic microorganisms. Recent studies have experimentally verified that *Acinetobacter* species, such as *A. baumannii* and *A. baylyi*, can assemble specific adhesins mediating initial interactions with host substratum, e.g., T4P, Ata, and Bap [45,47]. The T4P of Gram-negative organisms are mainly composed of a major pilin subunit protein PilA, outer membrane secretin pore PilQ, and inner membrane platform protein PilC, which interacted with three ATPases PilB/PilT/PilU mediating fiber extension and retraction. As a central part of competence-induced DNA uptake machinery, T4P has been found important for twitching motility and natural transformability of *A. baumannii* [57]. Here, the pangenome of *A. nosocomialis* possessed all the genetic elements (21 genes) responsible for the biosynthesis of the T4P system, except for the gene *pilO* of *A. baumannii*. The motor protein PilB driving the T4P extension is single and highly conserved in the genomes of all *A. nosocomialis* strains (Figure S5), and the corresponding gene product (570 aa) encoded by OG_4222 shares 98.9% identity with the homolog (570 aa) of *A. baumannii* strain ACICU. Several genes encoding protein variants were also found in the pangenome of *A. nosocomialis*, e.g., *pilQ*, *pilA*, and *pilE*. Two variants of *pilQ* encoding T4P secretin (OG_5546, 721 aa; OG_4252, 708 aa) were identified in the 139 and 18 strains, respectively (Table S6). Both variants of *A. nosocomialis* PilQ share 97.1% and 87.5% identities with the PilQ homolog (VFG050372, 686 aa) of *A. baumannii* ACICU, which is responsible for the translocation of the pilus to the cell surface [57]. Besides, the major pilin protein PilA is represented by five variants, all of which contain a pilin domain (FP00114) and prokaryotic N-terminal methylation motif (PF07963). Notably, the gene *ata* (OG_9377) encoding a trimeric autotransporter adhesin, which could express lectin activity by binding host glycans during adherence of *A. baumannii* [58], was absent in most *A. nosocomialis* strains, but only present in the three strains of ST1715.

Clinically relevant *Acinetobacter* strains can form biofilms that are an important pathogenic factor contributing to both device- and non-device-associated nosocomial infections [14,59]. In the pangenome of *A. nosocomialis*, we also identified 36 genes encoding the proteins responsible for the production of several virulence factors associated with biofilm formation, including AdeFGH efflux pump, Csu fimbriae, alginate, type 3 fimbriae, and Poly-β-1-6-*N*-acetylglucosamine (PNAG) (Table S6). Of these genes, about three-quarters are highly conserved and present in most strains. Intriguingly, the core genes, i.e., *adeFGH* (OG_1022/OG_541/OG_3378), of *A. nosocomialis* encode the proteins sharing 98.0%, 99.7%, and 98.5% identity with *A. baumannii* AdeFGH, which constitute an efflux pump participating in the transport of autoinducer molecules during biofilm formation [60]. Besides, *A. nosocomialis* could synthesize type 3 fimbriae-like cell surface appendages, according to the presence of six gene-encoding proteins showing homology with *K. pneumoniae* MrkBCD [61] (Table S6). Except for the structural complex mentioned above, the single gene *ompA* encoding outer membrane protein A, which can facilitate the persistence and survival of *A. baumannii* by assisting biofilm formation on abiotic surfaces [62], was detected in all the strains of *A. nosocomialis* (Figure S6). Two variants (OG_2713, 349 aa; OG_5230, 342 aa) of *ompA* code for the proteins share 91.4% and 86.2% identity with *A. baumannii* OmpA (356 aa), which has been found to play a role in adherence and invasion into host cells during infection [63].

## 5. Conclusions

In this study, a highly abundant pathogen, *A. nosocomialis*, was uncovered using the metagenomics data from a sepsis patient with a wound infection. We compared and evaluated three distinct strategies for genome reconstruction of an *A. nosocomialis* ST410 strain. A good assembly with 100% genome completeness was yielded for characterizing the phylogenetic and epidemiological features of the metagenomic strain WHM01. Furthermore, the population genomic analyses uncovered the genes encoding a number of virulence factors in *A. nosocomialis*, which should provide a foundation for future research into functional characterization of their roles in the pathobiology of this nosocomial pathogen. Our work reveals that metagenomic surveillance may be a promising application for promoting the pathogenic risk of any bacterial strains dominating the microbial community, whilst decoding genetic diversity of virulence genes and/or other genotypes of interest in clinical settings.

## Figures and Tables

**Table 1:** Summary of incidence and abundance of *A. nosocomialis* present in the healthy human samples from five body sites ^a^.

Body Site	No. of Samples	*A. nosocomialis*			
		Incidence	Relative Abundance (%)		
			Mean	Max	SD
Skin	363	18 (4.96%)	0.0609	0.3585	0.1120
Oral	740	1 (0.14%)	0.0002	0.0002	-
Stool	12998	7 (0.05%)	0.1298	0.8184	0.3050
Nasal cavity	93	0	-	-	-
Vagina	96	0	-	-	-

^a^ The statistics displayed herein are calculated based on the metagenomic resources retrieved from the curatedMetagenomicData package [23].

**Table 2:** Comparison of genome assemblies of an *A. nosocomialis* strain recovered from the metagenome.

	Assembly I	Assembly II	Assembly III
No. of contigs			
>500 bp	226	103	123
>10,000 bp	87	63	62
N50 (bp)	40,596	79,429	82,591
NGA50 (bp)	31,762	45,950	50,420
Largest (bp)	173,266	172,967	173,444
Total (bp)	3,679,592	3,873,672	3,892,781
GC (%)	38.84	38.69	38.77
Genome fraction (%) ^a^	86.72	87.00	87.38
Completeness (%)	99.86	100	100
Contamination (%)	0.14	0	0
No. of CDSs	3497	3696	3704
No. of rRNAs	2	3	8
No. of tRNAs	57	58	67

^a^ The reference genome used for the metric is from *A. nosocomialis* strain M2 (CP040105).

## Data Availability

The metagenomic sequencing data comprising microbial reads has been deposited at the NCBI SRA database, under BioProject accession PRJNA824592. The genome sequence of *A. nosocomialis* WHM01 has been deposited at the NCBI GenBank, with the accession JALOCQ000000000.

## Supplementary Materials

The following supporting information can be downloaded at: https://www.mdpi.com/article/10.3390/pathogens11080838/s1. Figure S1: Scatter plot of percentage relative abundances of *A. nosocomialis* detected in three body sites of healthy individuals. Figure S2: Whole-genome phylogeny and metadata of all *A. nosocomialis* strains, based on the sequence alignments of core SNPs (A) and core genes (B). Figure S3: Phylogenetic tree of the MLST loci-sequences of 155 *A. nosocomialis* strains. Two strains are excluded from the MLST tree, due to the absence of the *rpoB* gene. Figure S4: Genetic distribution and sequence conservation of accessory genes encoding products involved in the capsule biosynthesis of *A. nosocomialis*. Figure S5: Genetic distribution and sequence conservation of accessory genes encoding products involved in the adhesion of *A. nosocomialis*. Figure S6: Genetic distribution and sequence conservation of accessory genes encoding products associated with exotoxins, immune modulation, and biofilm formation of *A. nosocomialis*. Table S1: Species-level taxonomic profile of the microbial community in the specimen. The organisms with relative abundance scores greater than 0.005% are shown herein. Table S2: The list of the ANI values between paired genomes of six *Acinetobacter* species. Table S3: Summary of metadata information of all the *A. nosocomialis* genomes used in this study. Table S4: Pairwise SNP distance matrix between any two genomes among 157 *A. nosocomialis* strains. Table S5: Protein functional annotation of the pangenome genes present in *A. nosocomialis*. Table S6: Summary of the predicted virulence-associated genes present in the pangenome of *A. nosocomialis*. The information relevant to the genes includes the classification of virulence factors, Pfam annotations of protein structural domains, gene identifiers of the metagenomic strain WHM01, and number of strains possessing the corresponding genes among all the strains tested. Table S7: List of BLAST score ratio values computed by searching the virulence-associated genes against all the genomes of *A. nosocomialis*.
