# Supplementary figures and images for: Metagenomic Insights into Pathogenic Characterization of ST410 Acinetobacter nosocomialis Prevalent in China
Source: Pathogens. 2022 Jul 27;11(8):838. doi: 10.3390/pathogens11080838 (PMC9414201; doi:10.3390/pathogens11080838)

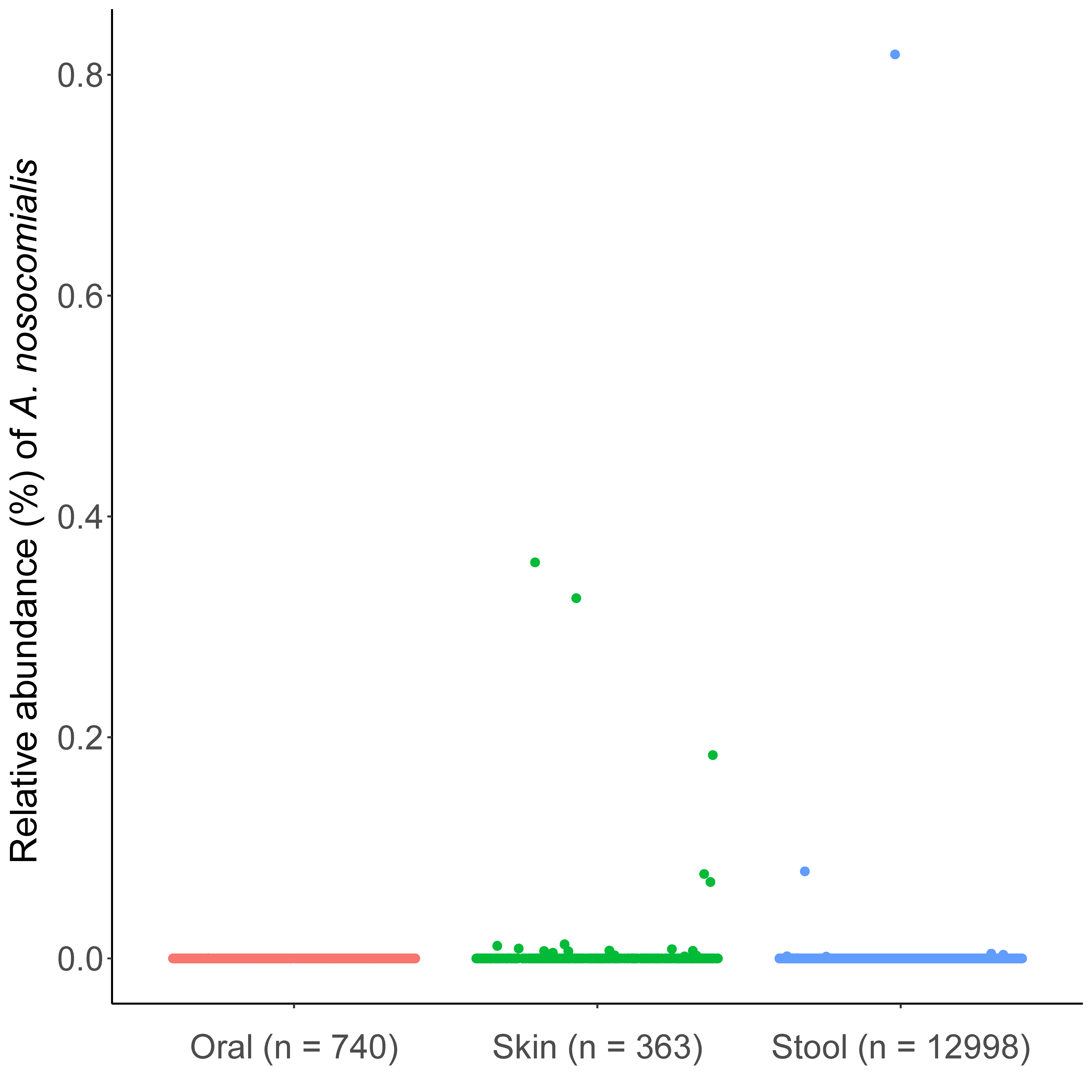

Supplement: Supplementary file 1 [file pathogens-11-00838-s001.zip › Figure S1.tiff]

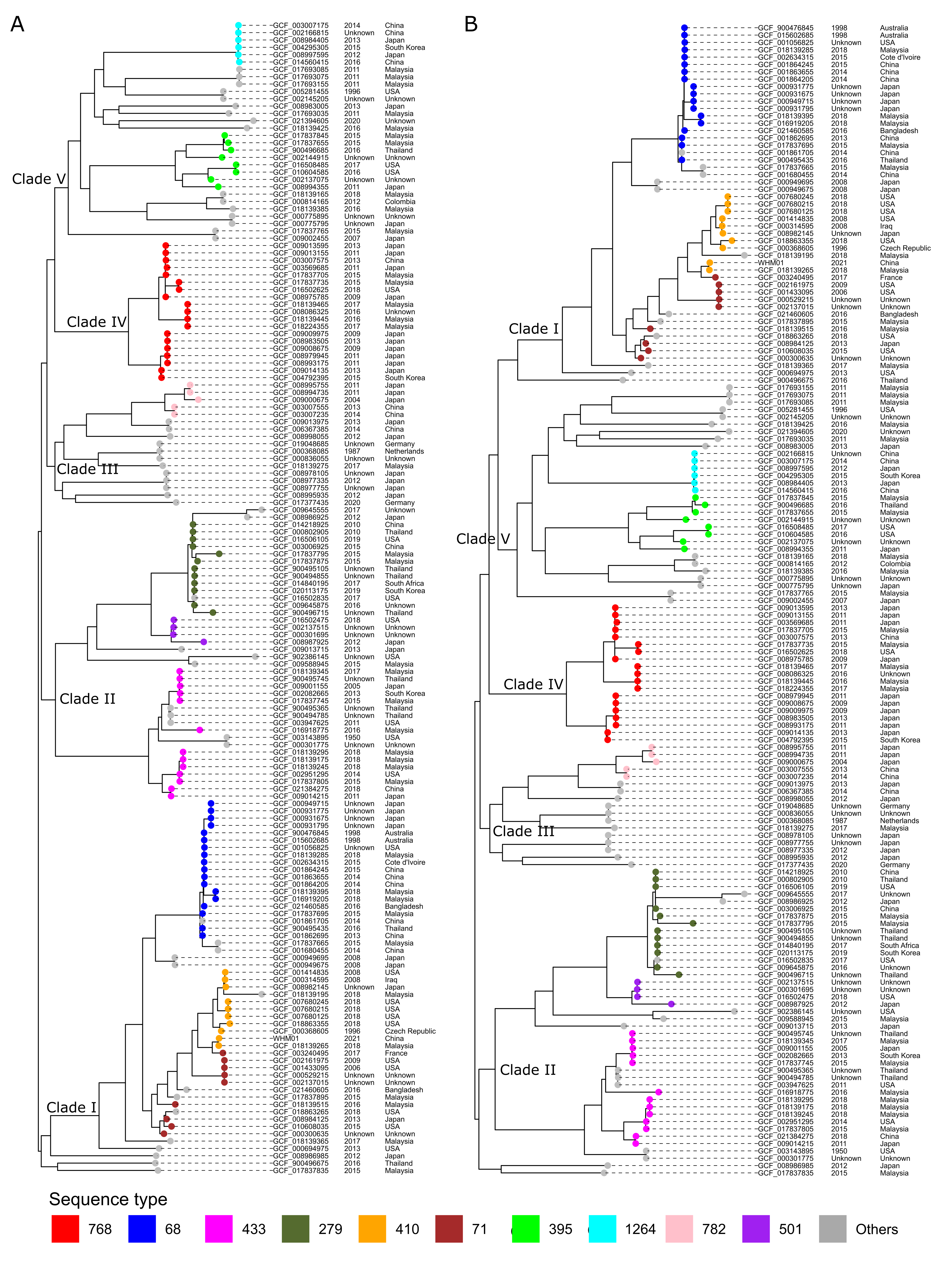

Supplement: Supplementary file 1 [file pathogens-11-00838-s001.zip › Figure S2.tif]

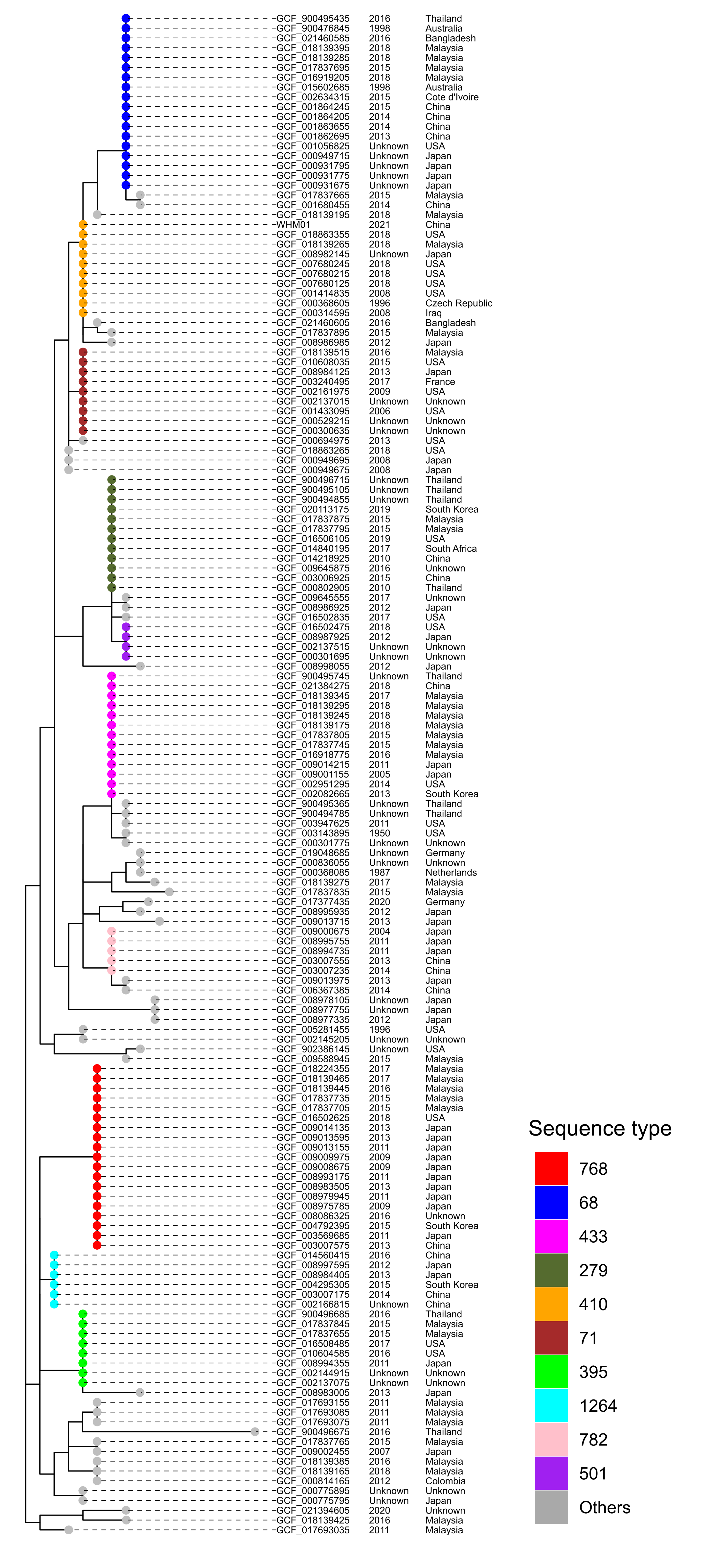

Supplement: Supplementary file 1 [file pathogens-11-00838-s001.zip › Figure S3.tif]

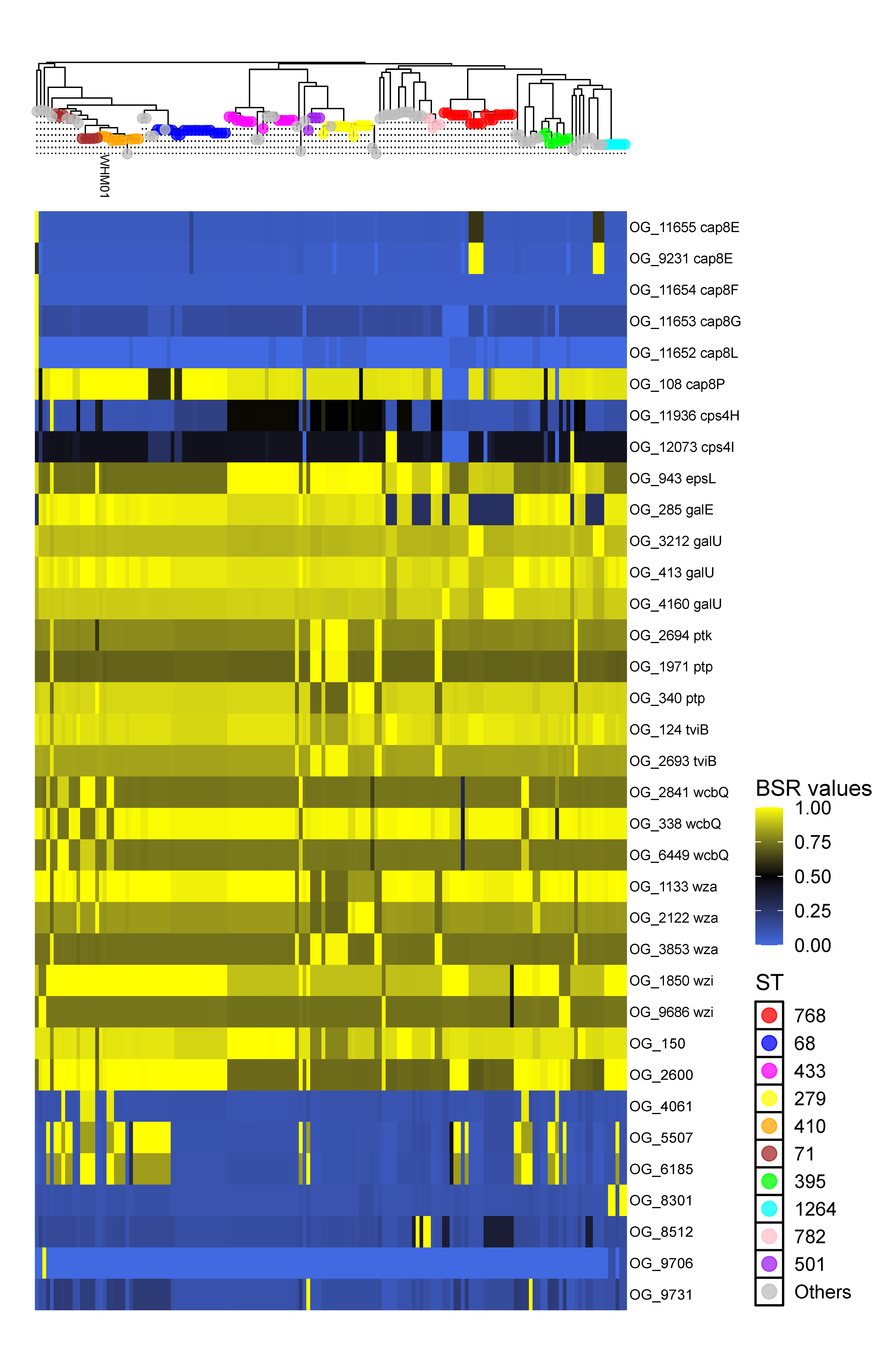

Supplement: Supplementary file 1 [file pathogens-11-00838-s001.zip › Figure S4.tif]

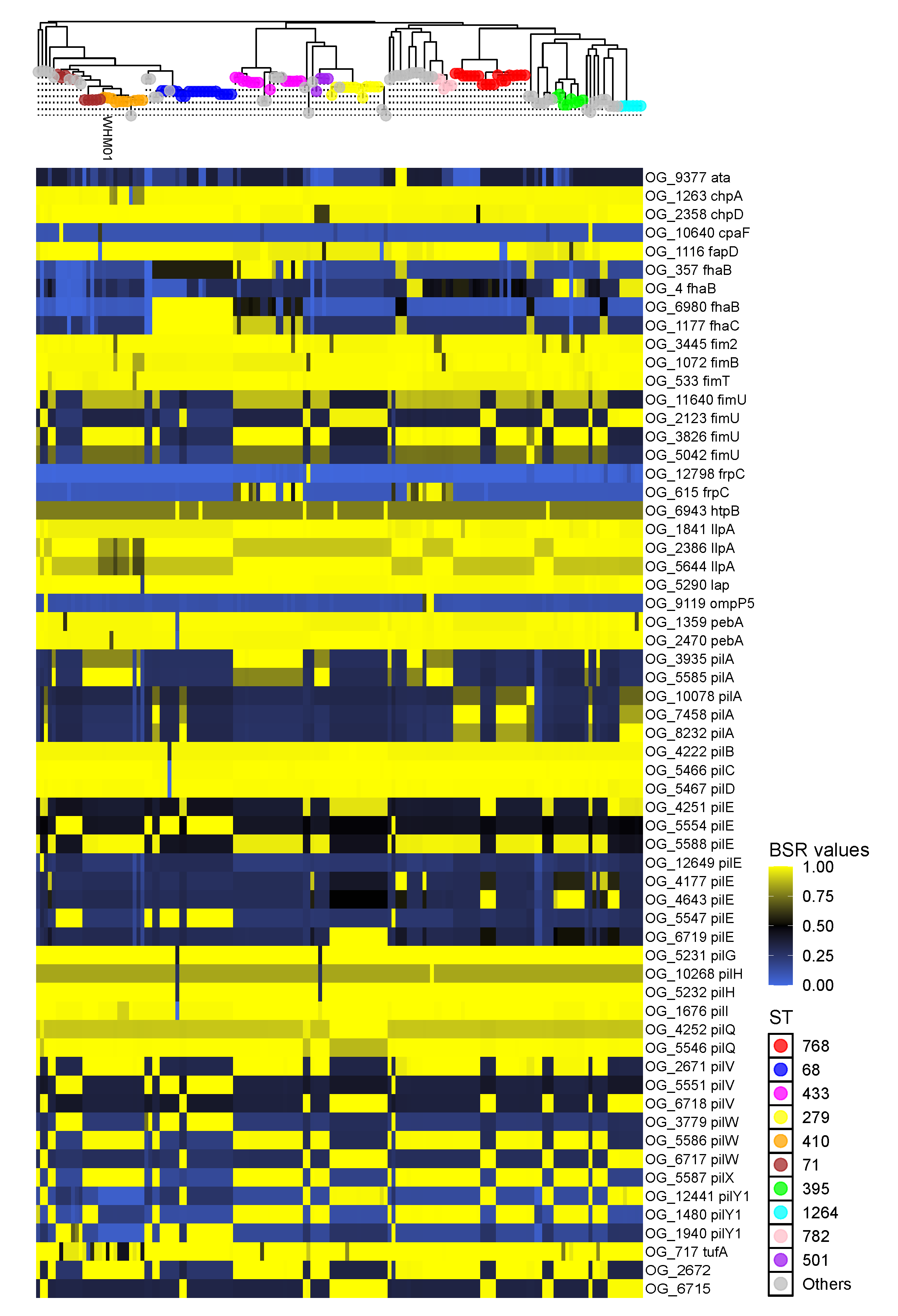

Supplement: Supplementary file 1 [file pathogens-11-00838-s001.zip › Figure S5.tif]

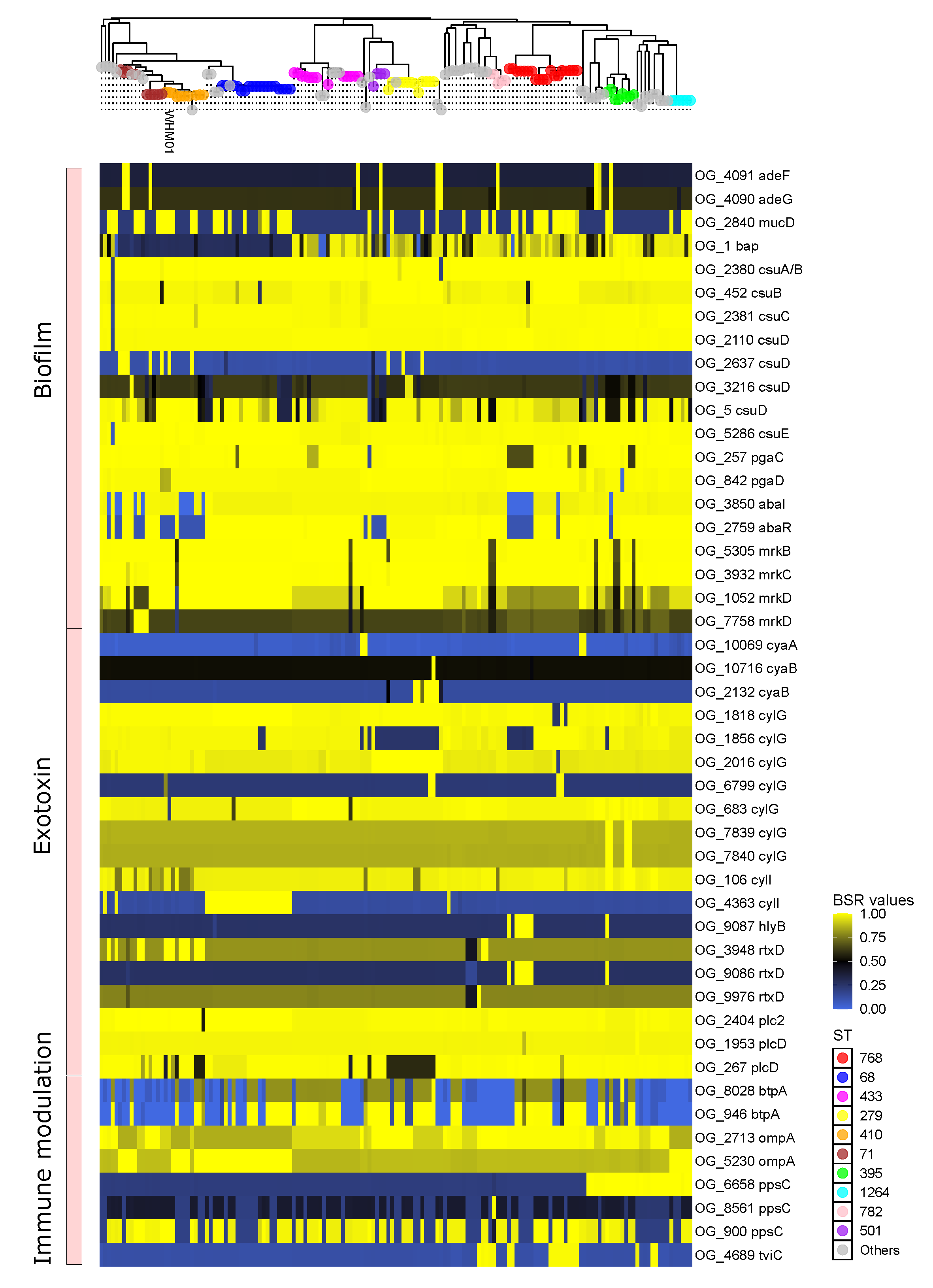

Supplement: Supplementary file 1 [file pathogens-11-00838-s001.zip › Figure S6.tif]
